# Supplementary material for: Loss of REP1 impacts choroidal melanogenesis and vasculogenesis in choroideremia
Source: Biochim Biophys Acta Mol Basis Dis. 2024 Feb;1870(2):166963. doi: 10.1016/j.bbadis.2023.166963 (PMC11157692; doi:10.1016/j.bbadis.2023.166963)
Supplement: Supplementary file 1 — Supplementary material [file mmc1.docx]

**Supplementary material**

**Zebrafish qRT-PCR primers**

| **Table S1: Zebrafish melanogenesis primer sequences** | | |
| --- | --- | --- |
| **Gene** | **Forward primer** | **Reverse primer** |
| ***mitfa*** | AGAAGCAGAAGTCAGAGCCC | CCGCCTTCTTCGTTCAATGAG |
| ***tyr*** | ATTTACAGGATCCAGGTCAGCG | GCGTGTCACATGACGTGTTT |
| ***pmela*** | CCAACGCAATCACTGGTGTA | GCAATGATTAGTGCGGCCTG |
| ***pmelb*** | GATCGTCCAGGTGGCTGTAG | TTCAGTGGGGATGCTTCCTTG |
| ***tyrp1a*** | CTGCCGTTCTGGGATTTCTC | GCTGGAGAAGATGGAGTTGGAG |
| ***dct*** | TCCATCGACCTCGAAGAACTG | GGCTCAAACTCGCGTTTTCT |
| ***actin*** | CGAGCTGTCTTCCCATCCA | TCACCAACGTAGCTGTCTTTCTG |


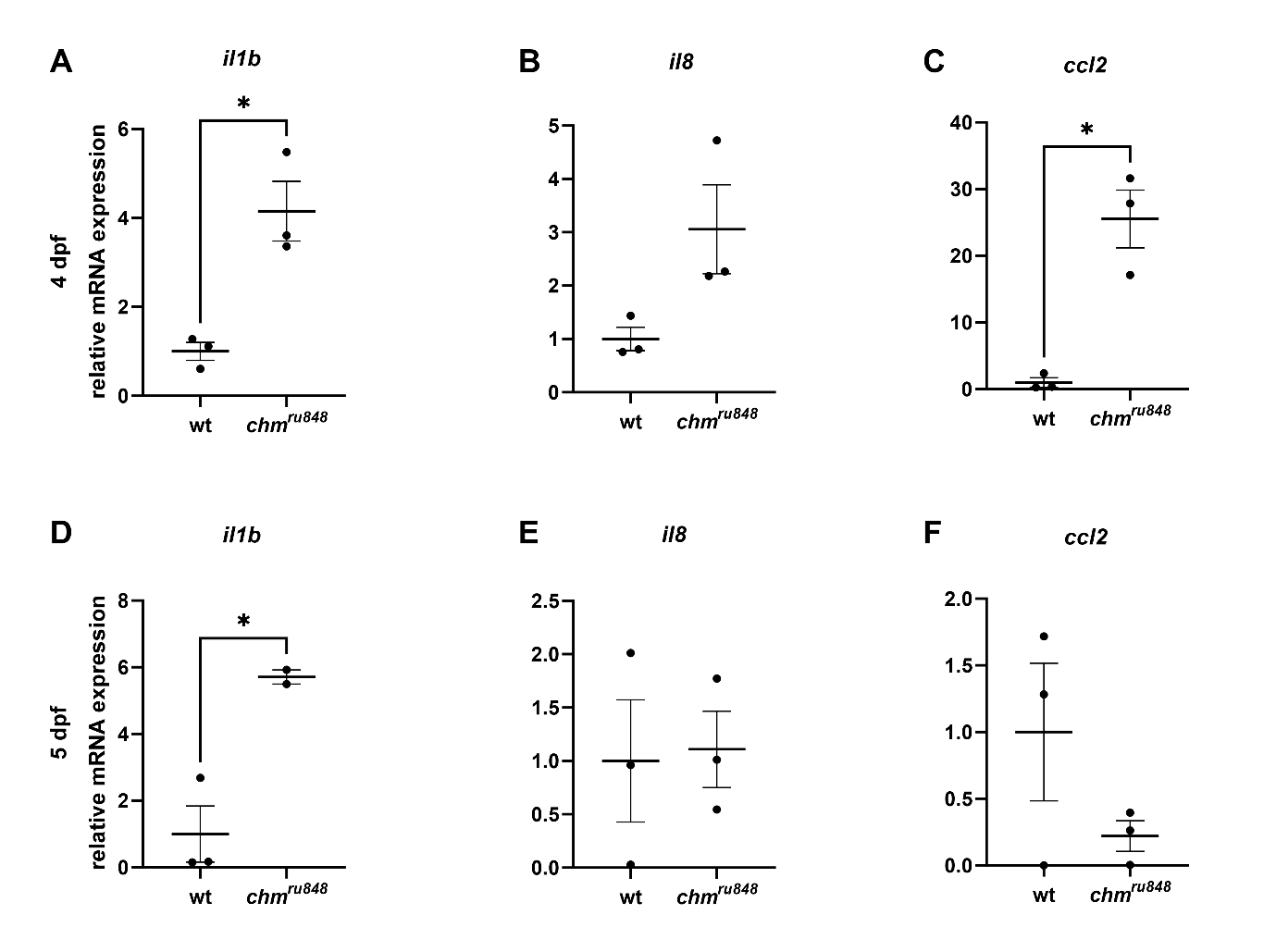


**Figure S1:** **Upregulation of inflammatory cytokines in *chm^ru848^* zebrafish**. Expression of inflammatory cytokines were analysed by RT-qPCR at 4 dpf (A-C) and 5 dpf (D-F) in zebrafish eyes. Data are expressed as mean±SEM from n=3. Statistical significance was determined using t-test. *p<0.05.


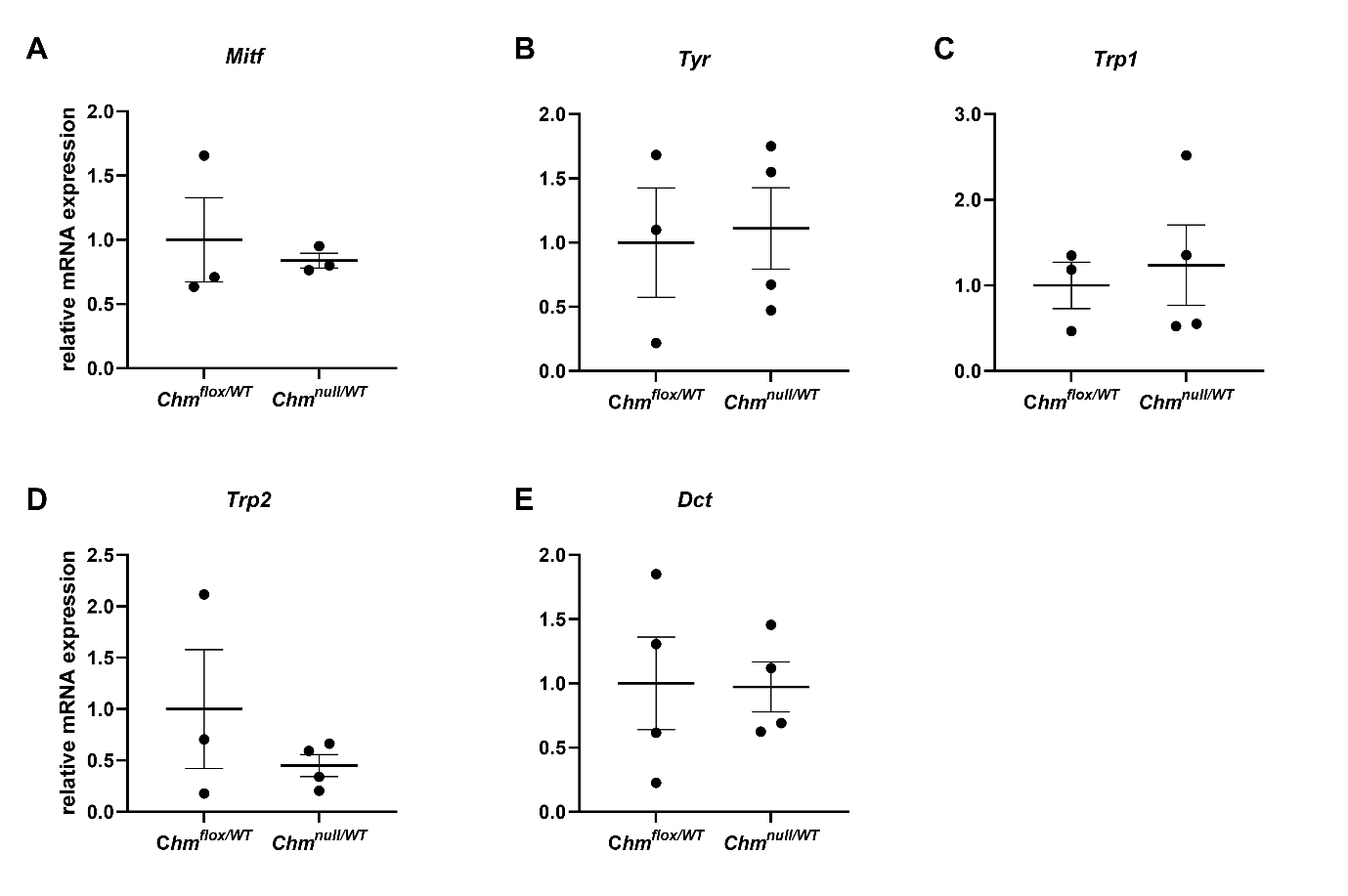


**Figure S2: Expression of melanogenesis genes remain unaltered in *Chm^null/WT^* mice.** (A-E) The expression of melanogenesis genes in *Chm^null/WT^* mice was not significantly different compared to the Wt (*Chm^flox/WT^*) although a decreasing trend was observed in *Mitf* and *Trp2* levels in *Chm^null/WT^* mice. Data are expressed as mean±SEM from n=4.
